# Propionic acidemia identified in twin siblings conceived by in vitro fertilization (IVF) with parents who were unknown carriers of a *PCCA* mutation

**DOI:** 10.1186/s12884-020-03391-z

**Published:** 2020-11-12

**Authors:** Ye Tian, Guojie Wang, Wujuan Shi, Xiaohong Bai

**Affiliations:** 1 Reproductive Medicine Center, Department of Gynecology and Obstetrics, Tianjin Medical University General Hospital, No.154, Anshan Road, Heping district, Tianjin, 300052 China; 2 Department of Neonatology, Tianjin Children’s Hospital, No.238, Longyan Road, Beichen District, Tianjin, 300134 China

**Keywords:** Propionic acidemia, *PCCA*, In vitro fertilization, Genetic carrier screening

## Abstract

**Background:**

Propionic acidemia (PA) is a severe monogenic disorder characterized by a deficiency of the mitochondrial protein propionyl-CoA carboxylase (PCC) enzyme, which is caused by mutations in the *PCCA* or *PCCB* gene. Preconception carrier screening could provide couples with meaningful information for their reproductive options; however, it is not widely performed in China.

**Case presentation:**

This report describes a case of dizygotic twin siblings conceived by in vitro fertilization (IVF) and diagnosed with propionic acidemia (PA). Their parents had no history of PA. Tandem mass spectrometry and urine gas chromatography/mass spectrometry (GC/MS) of the twin siblings revealed markedly elevated propionyl carnitine (C3), C3/C2, and 3-hydroxypropionate in the plasma and urine. Whole-exome sequencing was performed for the twin siblings. A homozygous missense mutation, c.2002G > A (p.Gly668Arg) in *PCCA*, was identified in the twin siblings. Sanger sequencing confirmed the homozygous mutation in the twin siblings and identified their parents as heterozygous carriers of the c.2002G > A mutation in *PCCA*. Both neonates in this case died. This is an emotionally and financially devastating outcome that could have been avoided with genetic carrier screening before conception. If couples are screened before IVF and found to be silent carriers, then reproductive options (such as preimplantation genetic diagnosis or prenatal diagnosis) can be offered to achieve a healthy newborn.

**Conclusion:**

This case is a reminder to infertile couples seeking IVF that it is beneficial to clarify whether they are silent carriers before undergoing IVF.

**Supplementary Information:**

The online version contains supplementary material available at 10.1186/s12884-020-03391-z.

## Background

Propionic acidemia (PA) is a rare, life-threatening metabolic disorder caused by a deficiency of the mitochondrial propionyl-CoA carboxylase (PCC) enzyme. PA has an estimated incidence of 1 per 100,000 to 150,000 [1]. The PCC enzyme catalyzes carboxylation of propionyl-CoA to D-methylmalonyl-CoA; therefore, PA is characterized by toxic accumulation of propionyl-CoA and metabolites of branched chain amino acid catabolism, such as 3-hydroxypropionic acid and propionyl carnitine (C3) in plasma and urine [1]. Most patients present with ketoacidosis, difficulty feeding, lethargy, seizures, encephalopathy, and failure to thrive during the neonatal period. Patients who live beyond the neonatal period may experience intellectual deficits, chronic kidney disease, cardiomyopathies, and pancreatitis [2, 3].

PCC is a heterododecamer composed of six propionyl-CoA carboxylase alpha subunits and six propionyl-CoA carboxylase beta subunits. The alpha and beta subunits of the PCC enzyme are encoded by the *PCCA* and *PCCB* genes, which map to chromosome 13q32.3 and chromosome 3q22.3, respectively. Mutations in *PCCA* or *PCCB* result in defects of the PCC enzyme, leading to PA. To date, more than 200 variants of the *PCCA* or *PCCB* genes have been identified [4, 5]. PA is inherited in an autosomal recessive manner. Missense mutations account for approximately 50% of the variations and are the most frequent defects in *PCCA* and *PCCB* [6].

Assisted reproductive technologies (ART), including in vitro fertilization (IVF), are used to overcome infertility in many families. The goal of IVF is not only to achieve pregnancy but also to take home a healthy baby. Mendelian diseases are individually rare; however, collectively, they account for approximately 20% of infant mortality cases and approximately 10% of pediatric hospitalizations [7, 8]. Preconception carrier screening has gained more recognition; however, it is not widely performed for ART patients in China. We report a case of dizygotic twin siblings conceived by in vitro fertilization (IVF) who died of PA. The homozygous missense mutation c.2002G > A (p.Gly668Arg) in *PCCA* was identified in the twin siblings using whole-exome sequencing. The parents had no history of PA but were unknown carriers of the c.2002G > A mutation in *PCCA*.

## Case presentation

Two newborn dizygotic twin siblings were born to unrelated nonconsanguineous parents with normal karyotypes. The pregnancy was achieved by IVF because of the mother’s fertility problems. The father had normal documented semen parameters. The mother initially presented to our institution at age 28 years with a 1-year history of secondary infertility. She had two previous pregnancies that resulted in one spontaneous abortion and one abortive ectopic pregnancy. Her menstrual cycles were irregular, with intervals of 28–45 days. She was diagnosed with polycystic ovary syndrome and hypothyroidism at our reproductive center. Her hysterosalpingogram showed no abnormalities, but she experienced a second ectopic pregnancy after ovulation induction treatment in our reproductive center. Unilateral salpingectomy was conducted through laparoscopy. The couple decided to try IVF. GnRH antagonist protocols were performed, 21 oocytes were retrieved, 16 oocytes were fertilized, and 12 embryos were formed; however, only four good-quality day 3 embryos were frozen. No pregnancies resulted from the first frozen embryo transfer (FET) cycle during which 2 day 3 embryos were thawed. The second FET cycle involved transferring 2 day 3 embryos, which resulted in the birth of dizygotic twin siblings. The mother was 31 years old and the father was 36 years old at the time of delivery.

The patients were dizygotic twin siblings born at 38 weeks of gestation. The birth weight of the girl was 2630 g and the birth weight of the boy was 2130 g. The girl was 1 week old when she presented with difficulty feeding, coma, seizures, acidosis, respiratory failure, neonatal septicemia, and hyperammonemia. A urinary organic acid analysis revealed elevation of 3-hydroxypropionic acid. Tandem mass spectrometry showed that she had elevated C3 and C3/C2 (Table 1), indicating she had PA. Her twin brother presented with difficulty feeding, lethargy, acidosis, neonatal septicemia, and hyperammonemia at the age of 1 week. Elevated 3-hydroxypropionic acid in urine and elevated C3 and C3/C2 in plasma suggested that he had PA (Table 1).

**Table 1:** Results of MS/MS for twin siblings

Case	MS/MS (blood)			
	C0 (10–50 μmol/L)	C3 (0–3.1 μmol/L)	C3/C2 (0.02–0.25)	C3/C0 (0.01–0.24)
Girl	6.819	14.581	2.871	2.138
Boy	8.377	18.994	2.261	2.267

MS/MS, tandem mass spectrometry

**Fig. 1:**
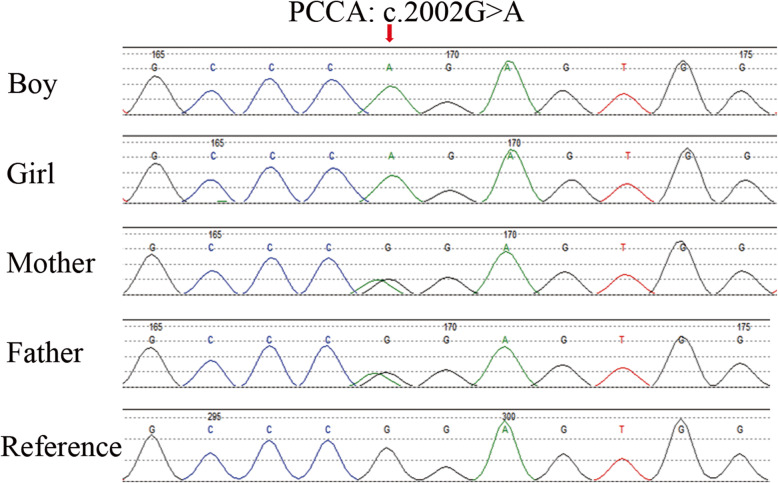
Sanger sequencing was performed to validate genotypes. Representative chromatograms of dizygotic twin siblings and their parents are shown. The arrows in the chromatograms indicate the location of the mutation (c.2002G > A)

To identify the cause, whole-exome sequencing was performed for the twins by Guangzhou Kingmed Diagnostics Group Co., Ltd. Blood samples were collected from the patients, and genomic DNA was extracted using the QIAamp DNA Blood Mini kit (Qiagen, Hilden, Germany). The DNA libraries after enrichment and purification were sequenced on the NextSeq500 sequencer according to the manufacturer’s instructions (Illumina, San Diego, CA, USA). All reads were aligned to the reference human genome (UCSC hg19) using Burrows-Wheeler Aligner software (version 0.5.9-r16). The homozygous missense mutation c.2002G > A (p.Gly668Arg) in *PCCA* was identified in both twins; c.2002G > A is a known pathogenic mutation leading to PA. Furthermore, bioinformatic tools including PolyPhen 2, Sift, Mutation Taster, and MutPred score all classified this variation as pathogenic (Table S1). Sanger sequencing was performed for the two patients and their parents (Fig. 1). The parents had no history of PA; however, they were both identified as carriers of mutation c.2002G > A in *PCCA*. The girl died 24 h after hospitalization. Her twin brother died at age 1 month.

## Discussion and conclusions

We report dizygotic twin siblings conceived by IVF who both died of PA soon after birth. Genetic testing showed that the twins had a pathogenetic mutation in the *PCCA* gene and that their parents were unknown carriers of that *PCCA* mutation, thus confirming the diagnosis of PA. This type of event is mentally, emotionally, and financially devasting for families.

Variation c.2002G > A is located in exon 22 and causes an amino acid substitution at the 668 site of the PCCA protein. Gupta et al. screened *PCCA* and *PCCB* genes of 25 children in India with PA and identified that the c.2002G > A variation was associated with PA [9]. Bioinformatic tools classified this variation as pathogenic. This missense mutation c.2002G > A is in the biotinylation domain at the C-terminus. Campeau et al. examined this mutation (as Gly643Arg) in vitro to determine its capacity to support biotinylation and found that it decreased biotinylation by 95% of its initial value [10]. In this case report, the infertile couple was heterozygous for the missense mutation c.2002G > A; however, their genetic compositions were unknown before the delivery of their twins with PA.

Preconception carrier screening revealed that the average genomic carrier burden for severe pediatric recessive mutations was 2.8 [11]. Currently, because of technological advances like next-generation sequencing (NGS), preconception screening has become more feasible regardless of family history. According to recommendations (American College of Obstetrics and Gynecology Committee on Genetics), the disorders included in expanded carrier screening panels should: have a carrier frequency of 1 in 100 or greater, have a well-defined phenotype, have a detrimental effect on quality of life, cause cognitive or physical impairment, require surgical or medical intervention, or have an onset early in life [12]. The overall outcome of PA remains poor despite the existence of effective therapy such as a low-protein diet [1]. PA could be predicted through the usage of preconception genetic carrier screening and genetic counseling about reproductive options (such as preimplantation genetic diagnosis or prenatal diagnosis).

In the present study, twins with PA were conceived by an infertile couple through ART. Expanded preconception genetic carrier screening using NGS found that 5% of couples undergoing ART using their own gametes had pathogenic variants [13]. Researchers have estimated that preconception genetic carrier screening before ART for couples and patients requiring gamete donation results in prevention of 1.25% of newborns from being affected [13]. Franasiak et al. conducted expanded carrier screening for 3738 infertile couples and identified eight couples at risk of having an affected child, which indicated that expanded carrier screening affected the clinical decisions in 0.21% of cases associated with the infertile population [14]. Preconception carrier testing, ideally before pregnancy, is recommended by various professional societies [12, 15]. It is important to perform genetic screening of couples before ART to determine any genetic mutations so that steps (such as PGD) can be taken so that newborns conceived with ART are not affected. However, preconception carrier screening is not widely performed for ART patients in China. This case should be a reminder to infertile couples that it is critical to be aware of whether they are silent carriers before undergoing ART to reduce the risk of having an affected newborn. If two silent carriers are screened before IVF, a PGD procedure will be offered to achieve a genetically healthy newborn [16]. Further large-scale studies were needed to evaluate the benefits and the necessity of preconception carrier screening for infertile couples seeking ART.

## Supplementary Information

**Additional file 1:** **Table S1.** PCCA variants identified in twin siblings with propionic academia.

## Data Availability

All datasets generated for this study are included in the article.

## Abbreviations

PA: Propionic acidemia
IVF: In vitro fertilization
PCC: Propionyl-CoA carboxylase
ART: Assisted reproductive technologies
FET: Frozen embryo transfer
PGD: Preimplantation genetic diagnosis
NGS: Next-generation sequencing
