# Supplementary material for: Propionic acidemia identified in twin siblings conceived by in vitro fertilization (IVF) with parents who were unknown carriers of a PCCA mutation
Source: BMC Pregnancy Childbirth. 2020 Nov 12;20:689. doi: 10.1186/s12884-020-03391-z (PMC7659086; doi:10.1186/s12884-020-03391-z)
Supplement: Supplementary file 1 — Additional file 1: Table S1. PCCA variants identified in twin siblings with propionic academia. [file 12884_2020_3391_MOESM1_ESM.docx]

**Table S1. PCCA variants identified in twin siblings with propionic academia**

| Case | Gene | Position | Exon | Mutation | Gene type | Amino acid change | Mutation type | Function | Reference |
| --- | --- | --- | --- | --- | --- | --- | --- | --- | --- |
| Girl | *PCCA* | 13q32 | Exon22 | c.2002G>A | Homozygote | p.Gly668Arg | Missense mutation | Diminished PCC activity | [17, 9] |
| Boy | *PCCA* | 13q32 | Exon22 | c.2002G>A | Homozygote | p.Gly668Arg | Missense mutation | Diminished PCC activity | [17, 9] |

PCC, propionyl-CoA carboxylase
